# Navigating the "mode effect": A comparison of online questionnaires and face-to-face interviews

**DOI:** 10.1016/j.heliyon.2025.e41742

**Published:** 2025-01-06

**Authors:** Flora M. Díaz-Pérez, Alan Fyall, Carlos Gustavo García-González, Xiaoxiao Fu, Gary Deel

**Affiliations:** a Facultad de Economía, Universidad de la Laguna, La Laguna, Spain; b Rosen College of Hospitality Management, University of Central Florida, USA

**Keywords:** Multi-mode survey, "Mode effect", Accommodation, National and state parks, CHAID algorithm

## Abstract

From a methodological perspective, the "mode effect," which refers to the phenomenon where different survey methods can yield different responses despite asking the same questions, presents a significant contemporary challenge. Existing solutions suggested in the literature, such as the implementation of multi-mode surveys, have their drawbacks as they may introduce bias that could impact respondent behavior. This study aims to identify distinct visitor segments within two large populations, assessing their patterns of visitation to both national and state parks. Additionally, we aim to evaluate the presence of the "mode effect" when concurrently conducting face-to-face and online surveys. The primary contribution of this research lies in confirming the persistence of the "mode effect," even when employing the same questionnaire for the same tourism product, during the same time frame, but among different populations. Furthermore, we propose various strategies to mitigate the "mode effect."

## Highlights

• The COVID-19 pandemic spurred online surveys for tourism segmentation, but they face challenges linked to the "mode effect."
• This paper proposes a novel approach to mitigate the "mode effect" using market segmentation analysis on two populations.
• This procedure differs from Dolnicar [17], replacing multi-mode surveys with segmentation of two distinct visitor groups.
• This procedure differs from Dolnicar [17], replacing multi-mode surveys with segmentation of two distinct visitor groups.
• Findings highlight "accommodation" as a key variable in analyzing tourist activities using the CHAID algorithm.
• Results suggest significant improvements in online survey effectiveness for future tourism research.

## 1 Introduction

The ongoing health challenges posed by the COVID-19 crisis have necessitated the adoption of online surveys, as opposed to the preferred face-to-face surveys, for collecting information about the tourist market. While online surveys offer several advantages, they also come with certain disadvantages that need to be addressed to ensure their effectiveness. One common drawback is the lack of sample representativeness, which can lead to the "mode effect" – a situation where different survey methods yield different responses despite asking the same questions [1]. Empirical studies have consistently shown statistically significant differences between face-to-face and online samples across various scales.

To tackle these issues, existing literature has primarily focused on the implementation of multi-mode surveys, allowing respondents to choose their preferred mode of completing the questionnaire [2,3,4,1]. As Dolnicar argues, given today's high internet adoption rates, sample composition is no longer a key problem [3].

The ongoing COVID-19 pandemic underscores the need for improved online data gathering techniques in visitor market segmentation. However, these online techniques present their own set of challenges for researchers. Therefore, this paper aims to propose effective strategies to overcome these challenges. Our innovative approach involves implementing different survey modes – online and face-to-face – to collect information on market segmentation for the same tourism product but from two distinct, large populations using the same questionnaire. This approach stands out from previous studies [3], which applied a multi-mode solution to the same statistical population. Our proposed procedure is relevant as it strives to neutralize the "mode effect" that may occur when respondents answer multi-mode surveys.

The primary contribution of this study lies in highlighting the importance of improving the implementation of online questionnaires. Our statistical analysis indicates that, despite our efforts to neutralize the "mode effect" by using two different large populations for the same tourism product, the face-to-face procedure exhibits higher representativeness, fewer omissions, and greater consistency than the online procedure. This underscores the need for enhancements in the online questionnaire implementation process. Simultaneously, natural protected areas have historically attracted tourists seeking to spend their leisure time. These areas offer not only pleasure but also various health benefits, both physical and mental [5]. The increasing demand for tourism in natural protected areas has prompted governments to closely monitor visitation [6,7].

To minimize bias related to visitor choice of interview mode, we conducted a comparative study between two different large samples – visitors to Canary Islands National Parks and Florida State Parks – focusing on the same tourism product, natural tourism. We assessed data quality using two criteria: the number of omissions (non-responses to questions) and contamination by two forms of response styles. This forms the core of our study.

## 2 Study objectives and hypotheses

Comparative studies of destinations are still limited in terms of scope, as only a few types of destinations and/or tourism activities were covered. Conducting comparative research in the field of tourism is indispensable for assessing tourism performance and enriching the tourism literature with empirical insights from diverse perspectives. Such research explores various aspects, including destination image [8,9,10,11], tourism products and services [12,13,14], and tourism impacts [15,16]. These comparisons facilitate the identification of both similarities and distinctions, which in turn inform scholars and destination managers about competitive dynamics and market opportunities. In this respect knowing the market segments corresponding to natural parks located in different territories is thus a crucial aim in studying the behavior of national and state park visitors.

This study goes further by gathering information on the structural differences found in markets that visit similar tourism products albeit from two different territories, implementing multi-mode surveys. The objectives of this study are thus as follows: (i) to determining the different segments that configure the visitors**'** market of the national and state parks of the two populations under study – Canary Islands and Florida visitors; (ii) to build a model where the most significant variable –the dependent one-in the segmentation is explained by a range of independent variables of diverse types: sociodemographic, economic and based on travel characteristics; (iii) compare the different results obtained using the same questionnaire for both statistical populations – Canary Islands and Florida visitors; (iv) contrast the results obtained when diverse mode of surveys are applied: face-to-face and online with the final objective of understanding whether the "mode effect" is fulfilled.

Based on the above objectives, the hypotheses proposed in this study are as follows.

H1 For the two populations – Canary Islands and Florida visitors – the variable that has the most significant relationship with the rest is accommodation.

H2 The range of independent variables will be highly divergent when diverse modes of collecting the information are applied, face-to-face or online.

H3 There are significant differences for the representativeness of the two modes of surveys: online and face-to-face.

H4 Online respondents have lower response rates than face-to-face ones.

H5 The extreme response style (ERS) is influenced by sociodemographic variables (age, place of residence, education level) for both modes.

H6 In comparing the consistence of the two surveys modes it will be found significant differences between the factors of traveling.

## 3 Literature review

### 3.1 Multi-mode surveys

Implementing multimode surveys, such as combining online and face-to-face methods, has its advantages and disadvantages. Starting with online surveys, they offer significant advantages. Firstly, they provide an easy way to reach potential respondents, simplify the interviewers' tasks, and can be deployed swiftly. The distribution of questionnaires is cost-effective, and data analysis can be performed promptly, as noted by Dolnicar et al. [3] and Zhang et al. [1]. Additionally, online surveys can help overcome the issue of socially desirable responses, which are more common in face-to-face settings due to the presence of an interviewer, as discussed by Dillman et al. [2]. Online formats also allow for the incorporation of multimedia elements like sound, pictures, animations, and videos, and can offer incentives such as monetary rewards. These incentives are proven to be particularly effective in increasing participation rates and reducing drop-out rates, as higher incentives typically lead to higher response rates [4]. Various studies have demonstrated that response rates increase with the amount of incentive offered [17,18,19,20,21].

Specifically, the methodological issues associated with the online mode of surveying also include several challenges [3,1]. First, the selection process often escapes the researcher's control, making selection probabilities unclear and unbiased estimation nearly impossible. Second, for populations that infrequently use the internet, under coverage becomes a significant concern. Third, self-selection within the sample can lead to selection errors. Fourth, the offer of incentives might prompt some respondents to complete the survey multiple times or provide inaccurate responses to hasten the process and qualify for rewards [4]. Fifth, in online surveys with monetary incentives, there is a tendency for poorer respondents to participate more than wealthier ones. Sixth, there are concerns about the perceived risks to confidentiality [22]. These challenges underscore why a multimode approach to surveying is recommended.

However, the application of a multi-mode survey procedure can introduce additional disadvantages. Firstly, online survey respondents are more likely to choose options listed at the beginning of a list due to a "primacy effect," while face-to-face survey respondents are more prone to the "recency effect," where they prefer options listed last [2]. Furthermore, a combined approach can lead to contamination issues where biases may influence respondents' choice of one response mode over another [2]. Dolnicar [3] identifies extreme response style (ERS) and acquiescence response style (ARS) as indicators of this contamination, signaling low data quality. Demographically, response styles vary; ERS tends to increase with age [23,24], and males are more likely to exhibit higher acquiescence than females [24]. Therefore, age and gender will be included as control variables in individual-level analyses. Similarly, education level and socio-economic status are also significant factors [23,25,26]. In this paper, we attempted to address the aforementioned disadvantages by administering the same questionnaire to two distinct, large populations of the same tourist product.

### 3.2 Response style

Ulitzsch et al. in their publication titled "The role of response style adjustments in cross-country comparisons," discuss the complexity of validating the Likert scale [27]. They argue that validation is complicated not only by differences in measurement approaches but also by how respondents perceive and use the scale's response options. These differences are referred to as response styles (RS). Liu et al. define RS as a source of measurement error associated with Likert scales used for measuring attitudes, which can lead to measurement bias [28].

Response styles (RS) can manifest in several forms, each favoring different categories of responses: 1) Extreme RS, which favors categories at the ends of the scale, 2) Midpoint RS, which favors categories in the middle of the scale, and 3) Acquiescent RS, which favors categories indicating agreement [27]. Weijters et al. in their article "Assessing Response Styles Across Modes of Data Collection," address the growing prevalence of cross-mode surveys by employing three methods of data collection: 1) paper and pencil questionnaires, 2) telephone interviews, and 3) online questionnaires [29]. They compare these modes in terms of acquiescence, disacquiescence, and extreme and midpoint response styles. To facilitate this analysis, they introduce a new method called RIRSMACS, which operationalizes multiple response styles. However, their study does not address the bias inherent in cross-country comparisons.

On the other hand, the country-specific differences in response styles (RS) are a widely discussed topic in the literature, with significant contributions from researchers [30,31,32]. Ignoring these differences can distort the psychometric properties of survey data [33]. For instance, Ulitzsch et al. focus on adjusting response styles to enhance the accuracy of cross-country comparisons [27]. Liu et al. in their study "Comparing Acquiescent and Extreme Response Styles in Face-to-Face and Web Surveys" analyze how different data collection methods affect acquiescent and extreme response styles (ERS) [28]. They conducted two surveys using the same questionnaire with different populations: one face-to-face and the other online. These surveys, using data from the American National Election Studies (ANES) for the year 2012, yielded the following results: 1) both acquiescent and extreme response styles appeared in both the face-to-face and online modes; 2) face-to-face respondents exhibited more extreme response styles than those responding via the web. The methodology employed by these authors is the "Latent Class Analysis approach." Finally, Zhang et al. in their publication titled "Though Forced, Still Valid: Psychometric Equivalence of Forced-Choice and Single-Statement Measures," propose the two-alternative forced-choice format as a solution to the problem of cross-cultural response style bias, presenting it as a valid alternative to traditional Likert scales [34].

## 4 Methodology

### 4.1 Description of variables

The variables considered for segmentation encompass a diverse range of factors, including travel characteristics (such as means of transportation used, accommodation, frequency of visits, duration of stay, and park visited), demographic factors (age, educational level, gender), economic indicators (spending within the park, income, occupation), and geographic variables (place of residence) (see Table 1). Furthermore, some variables have been included to gauge the value attributed to service provision (see Table 3). Both Table 1, Table 2 provide an overview of the various categories within each variable and highlight the significance of differences between Canary Islands and Florida for each variable.

**Table 1:** Socio-demographic variables (∗chi-sq. for significant differences).

*Variables*	*Chi – sq. (p-value)*	*Categories*		*Percentages*	
				CNP	FSP
**GENDER**	0.637	(1) Male (2) Female		58.8 41.2	55.5 44.5
**AGE_GR**	**0.000∗**	(1) 18–35 years (2) 36–55 years (3) More than 55 years		29.0 37.1 33.9	66.4 29.4 4.2
**PLACE_RESIDENCE**	0.246	**CNP** (1) Spain (2) Rest of Europe and International	**FSP** (1) Florida (2) Rest of U.S.A. and International	37.1 **62.9**	30.6 **69.4**
**ANNUAL_INCOME**	**0.000∗**	(1) Low: <35000 € (CNP) or $ (FSP) (2) Medium: 35000–75000 € (CNP) or $ (FSP) (3) High: >75000 € (CNP) or $ (FSP)		54.4 28.7 16.9	26.6 47.3 26.1
**STUDIES**	**0.000∗**	(1) Basic or primary studies (2) High school diploma (3) Degree or postgraduate degree		4.7 40.6 54.7	1.2 13.3 85.5
**OCCUPATION**	**0.000∗**	(1) Unemployed, Homemaker or Student (2) Employee or Civil servant (3) Self-employed (4) Retired		10.6 54.9 13.5 21.0	12.5 71.9 14.1 1.5

∗ p-value <0.05 (Significant differences).

**Table 2:** Factors of travel (∗chi-sq. for significant differences).

*Variables*	*Chi – sq. (p-value)*	*Categories*		*Percentages*	
				CNP	FSP
**NATIONAL/STATE PARK**	**0.000∗**	**CNP** (1) Garajonay (2) Caldera de Taburiente (3) Teide (4) Timanfaya	**FSP** (1) Florida Keys (FK) (2) Honeymoon island (HI) (3) Marjorie Harris (MH)	9.7 8.4 70.0 11.9	32.6 33.2 34.3
**FREQUENT_VISITOR**	**0.016∗**	(1) Non frequent (Two visits or less) (2) Frequent (More than two visits)		68.3 31.7	82.9 17.1
**DURATION_STAY**	0.097	(1) Less than 2 h (2) Between 3 and 6 h (3) More than 6 h		17.1 67.4 15.4	12.1 60.4 27.5
**MEAN_TRANSPORT**	**0.000∗**	(1) Own car (2) Rent a car (3) Coach-Shuttle, Taxi, Public Transport, Bicycle, On Foot (4) Motorbike		17.8 60.6 18.6 3.0	65.3 26.3 7.9 0.5
**ACCOMMODATION**	**0.017∗**	(1) Hotel (2) Rented apartment, house or farmhouse (3) Own house or family/friend house (4) Camping, cruise and others		39.7 33.8 20.2 6.3	53.1 14.7 25.9 6.3
**SPEND_WITHIN (the park)**	0.094	(1) Low: Less than 10 € (CNP) or 60 $ (FSP) (2) Medium: 10–30 € (CNP) or 60–165 $ (FSP) (3) High: More than 30 € (CNP) or 165 $ (FSP)		52.3 27.1 20.6	50.6 17.3 32.2

∗ p-value <0.05 (Significant differences).

**Table 3:** Rating variables of the services and facilities of the Natural Parks using a Likert scale: (1) Very bad, (2) Bad, (3) Normal, (4) Good and (5) Very good.

SERVICES										
Variables and percentages	Very bad		Bad		Normal		Good		Very good	
	CNP	FSP	CNP	FSP	CNP	FSP	CNP	FSP	CNP	FSP
**RESTAURANTS**	0.0	3.4	4.4	5.5	27.8	18.2	48.9	42.3	18.5	30.5
**CAFES**	1.4	3.0	6.4	7.8	29.7	22.1	43.4	40.1	19.2	27.0
**VISITORS’ CENTRE**	0.0	1.0	0.6	4.4	10.8	16.1	45.0	39.4	43.6	38.9
**ACCOMMODATION**	0.0	2.4	0.0	5.5	23.7	20.5	36.8	37.3	39.5	34.4
**SHOPS**	1.4	3.0	10.1	5.9	37.7	22.6	31.9	36.6	18.8	31.9
**SECURITY**	14.7	1.6	11.8	5.9	14.7	23.0	32.4	36.8	26.5	32.8
**MAINTENANCE**	0.7	1.3	1.4	4.2	4.1	12.7	32.4	35.9	60.8	45.9
**VEHICLE**	1.9	1.7	1.1	6.9	10.2	20.7	37.5	34.7	49.2	36.0
**GUIDES**	1.1	1.8	6.5	4.4	6.5	14.4	34.8	31.5	51.1	45.6
**SHELTER**	2.5	1.5	12.5	7.7	22.5	19.9	25.0	34.4	37.5	36.5
**INFORMATION**	1.4	0.9	7.4	3.5	14.5	11.7	35.3	32.8	41.0	51.1
**OTHER SERVICES**	2.3	4.2	0.0	4.4	20.7	19.0	26.4	33.4	49.4	39.1
*FACILITIES*										
**PARKING**	5.0	1.1	12.6	3.3	33.3	16.4	32.0	36.5	17.1	42.7
**SIGNPOSTING**	1.5	0.7	3.6	2.9	11.4	11.9	40.0	41.3	43.6	43.3
**PATHS**	0.0	0.8	1.4	3.0	11.5	13.1	47.9	36.7	39.2	46.5
**ACCESSIBILITY**	15.8	2.3	21.1	6.4	21.1	15.4	21.1	36.1	21.1	39.8
**OTHER FACILITIES**	0.0	4.6	0.4	4.2	4.3	18.2	12.4	37.7	82.9	35.2

The key variables of utmost importance for this study encompass demographic and economic factors, alongside those linked to the nature and composition of visitors' trips to the national and state parks. Economic variables encompass the occupation of visitors and their expenditures within the park. Demographic variables include the age and nationality or country of residence of visitors. Additionally, variables related to the frequency of visits, duration of stay, type of accommodation establishment, means of transportation, and specific parks visited have been incorporated, as outlined in Table 2.

To assess whether there is a significant relationship or association between the categorical variables and the two populations under study, Pearson's Chi-square test was selected. Prior to applying the test to the variables in Table 1, Table 2, we ensured that the key assumptions were met [35]: the variables use a categorical measurement scale (nominal or ordinal), the categories are mutually exclusive, observations are independent, the sample size is sufficient, and each expected cell count is 5 or more. If any of these assumptions were not met, alternative statistical tests, such as Fisher's exact test or McNemar's test, would have been considered.

First, all variables—both sociodemographic and travel-related—are categorical, with categories clearly defined in Table 1, Table 2 About half of the variables are nominal, and the other half are ordinal, making them suitable for the test. The second assumption is also met, as the categories are mutually exclusive, ensuring no individual can belong to more than one category within the same variable. Third, the observations are independent since they pertain to two distinct populations. The sample sizes are large enough to meet the theoretical requirements of the Chi-square test. Finally, the expected frequencies for all cells are greater than 5, except for the "International" category under Place of residence, which was merged with a previous category to satisfy the assumption.

### 4.2 Data collection

National and state parks are significant attractions in destinations worldwide, drawing millions of visitors annually. In the case of countries like the USA and Spain, where cities and coastal destinations often take the spotlight, national and state parks tend to be overlooked, despite being iconic attractions for both nations [36,37,38]. Understanding the composition of their tourist demographics, identifying tourist trends, and analyzing patterns of tourist behavior is of paramount importance, especially in the context of contemporary issues like overtourism [39,40] and the growing emphasis on implementing more sustainable visitor management practices in national and state parks [41,42]. Managing national and state parks is particularly crucial in mass tourism destinations, with the Canary Islands in Spain and Florida in the USA being two major global destinations. Although these destinations may not carry the same iconic status as Florida's world-famous theme parks or the Canary Islands' popular beach resorts, natural protected area tourism plays a vital role in both, with numerous studies conducted in each region [43,44,45]. Building upon this existing research foundation, this study aims to delve into the visitation patterns of national parks in the Canary Islands, Spain, and state parks in Florida, USA. The study employs various methodologies to collect quantitative data through surveys directed at two distinct statistical populations experiencing the same tourism product. The Ethics Committees at the authors' research institutions confirmed that informed consent was obtained from all participants.

#### 4.2.1 Canary Islands' national parks (CNP)

This study is empirical and quantitative in nature. Visitors to the Canary Islands National Park (CNP) were interviewed face-to-face using a structured questionnaire, which was divided into two sections. The first section of the questionnaire focused on gathering information about the visitor's trip and their activities within the CNP. This included details such as the duration of their stay in the CNP, the type of accommodation they utilized, and the means of transportation they used to reach the park and move around inside it. The second section of the questionnaire consisted of questions aimed at collecting descriptive information about the interviewees themselves. These questions covered aspects such as age, gender, level of annual income, occupation, and other relevant demographic details. To ensure the accuracy of the data collected from park visitors, an ad-hoc survey was conducted during the period from October 2018 to February 2020. The ad-hoc survey provided a detailed insight into the demographics of the park's visitors, showing that: (1) Visitors came from over 20 countries, predominantly Germany and Britain, which made up the largest groups of non-resident visitors; (2) Local tourists constituted about one-third of the total visitor count. The interviewers were selected for their fluency in languages commonly spoken by visitors to Canarian National Parks, such as English, Spanish, German, French, and Russian, ensuring that respondents could communicate in their preferred language. Additionally, the interviewers received specialized training to enhance their survey effectiveness. For example, in group visits, a representative was chosen for the interview, and the distribution of interviews was adjusted according to the typical visitation rates at different points in the park.

#### 4.2.2 Florida's state parks (FSP)

To gather precise information about visitors to Florida's state parks, the same questionnaire used for the Canary Islands National Park (CNP) was employed. The survey was conducted online from December 2018 to January 2019, yielding precise insights into park visitors. Participants for the study were recruited using Amazon Mechanical Turk (MTurk), a widely used platform for sourcing research subjects [33]. MTurk is recognized as a reliable and cost-effective method for collecting data that is representative of behavioral sciences research. Its use extends beyond social science research, encompassing various fields including marketing, hospitality and tourism, psychology, political science, communication, and sociology [46].

### 4.3 Comparative quantitative design

Based on [47,48], quantitative designs can generally be classified as either experimental or non-experimental. In experimental designs, the researcher actively controls and manipulates one or more independent variables to observe their effect on dependent variables, allowing for the establishment of causal relationships. In contrast, non-experimental designs involve no manipulation of variables; the researcher observes and measures variables as they naturally occur, identifying relationships or associations without inferring causality.

Within experimental designs, classic experimental designs involve random assignment of participants to groups (e.g., experimental and control groups), while quasi-experimental designs lack random assignment.

Comparative designs, which are broader, may fall under quasi-experimental designs if they involve comparing groups after an intervention (e.g., two-group posttest-only design). They are also common in non-experimental designs, where groups are compared without any intervention—whether in descriptive studies (describing characteristics of a population) or correlational studies (examining relationships between variables without manipulation).

For one of the study's objectives (outlined in Section 2), a non-experimental comparative design is suitable. However, to achieve the other three objectives, a quasi-experimental design is required.

Initially, we used a non-experimental comparative design to compare visitors at two national parks using the same questionnaire in different locations. This allowed us to identify significant differences in sociodemographic characteristics, travel factors, visiting behavior, and other variables based on numerical data and statistical analysis, minimizing subjectivity.

To address the main objectives, we employed a quasi-experimental design. Here, the researcher introduced an intervention by assigning different data collection methods to each location: face-to-face interviews at CNP and online questionnaires via Mturk at FSP. While this involves manipulation, there was no random assignment or control group, thus classifying it as quasi-experimental. The aim is to compare the impact of these different data collection methods to determine whether the method itself influences the results.

### 4.4 Sampling technique

For the CNP surveys conducted between 2018 and 2020, a two-phase approach was utilized. The first phase took place during the high season in winter, including the Easter period. The second phase occurred in late spring and early summer, which corresponds to the low season in the Canary Islands. Participants were selected using systematic random sampling and interviewed on-site at various locations within the Canary Islands National Park. This sampling technique involves selecting every nth visitor at predetermined visit points for interviews, ensuring a systematic approach to participant selection.

To achieve a maximum estimation error of 3.5 % while considering the latest published number of visitors to the National Parks of the Canary Islands (7,413,123 visitors for 2017) [49], a sample size of 784 individuals was required. Ultimately, a sample of 866 tourists in the National Parks was obtained.

Regarding Florida's State Parks (FSP), over 32 million residents and visitors enjoyed these parks and trails in 2017 [50]. To ensure individual representativeness for each of the three most visited parks, surveys were conducted at the Florida Keys Overseas Heritage Trail, Honeymoon Island State Park, and Marjorie Harris Carr Cross Florida Greenway, attracting a total of 3,569,201 visitors. The total number of surveys needed to ensure representativeness for each park and the overall visitor population was determined to be 1066. This study used the online platform Amazon MTurk to collect a total of 1109 valid surveys. Since participants on MTurk voluntarily decide whether to participate, the resulting sample is non-probabilistic and based on convenience. To counteract this limitation, researchers can use demographic information from prior studies or population censuses to better represent the target population [51]. In this case, quotas were set for parks visited, gender, age, and place of residence to improve the sample's representativeness.

### 4.5 Two different modes of collecting information

Two distinct modes of data collection were employed: online for the Florida State Parks (FSP) and face-to-face for the Canary Islands National Park (CNP). This divergence in data collection methods necessitates a comprehensive comparative analysis of the effectiveness of both systems. To facilitate this comparison, the study adopted the same criteria proposed by Dolnicar, which encompass the following dimensions: 1) Representativeness: Comparing the socio-demographic profiles of respondents to assess the representativeness of the samples; 2) Data Quality: Assessing the quality of data, measured by the number of omitted items and the presence of data contamination, including extreme response style (ERS) and acquiescence response style (ARS); and 3) Consistency and Data Comparability: Analyzing the consistency and comparability of data through responses to questions [3].

To examine representativeness, the study conducted an analysis of significant differences in socio-demographic variables between the two destinations (p < 0.05). Additionally, for assessing data consistency, the study explored the relationship among travel factors and corresponding significance (p-value) for the chi-square statistic for both Canary Islands and Florida.

To study contamination levels, the study focused on questions related to visitors' self-evaluation of park services and the state of natural resource conservation, utilizing Likert scales with 5 possible responses. Likert scales with five response options are known to be susceptible to response style manifestations [52]. Cronbach's study concluded that concerning potential response style contamination in the two data sets, the levels of both ERS and ARS are low in both samples. Furthermore, no systematic differences were detectable between the online and paper survey samples.

To compare response styles, the study employed the following indicators (ranging from 0.00 to 1.00).

• Extreme Response Style Negative (ERS -): Proportion of questions receiving a (1) response.
• Disacquiescence Response Style (DRS): Proportion of questions receiving a (1) or (2) response.
• Middle Response Style (MRS): Proportion of questions receiving a (3) response.
• Acquiescence Response Style (ARS): Proportion of questions receiving a (4) or (5) response.
• Extreme Response Style Positive (ERS +): Proportion of questions receiving a (5) response [53].

### 4.6 Statistical analysis techniques used for both population

Initially, we compared the profiles of visitors from the two studied populations (CNP and CSP) using the indicators obtained from the same questionnaire, applying the Chi-square test to identify differences between the categorical variables. Next, we analyzed the two data collection methods (online for FSP and face-to-face for CNP) through several comparisons. This included an analysis of response styles in both samples, using the non-parametric Mann-Whitney test to detect significant differences, as well as a comparison of the visitor segmentations for CNP and FSP.

Market segmentation in tourism studies has often relied on regression methods [54,55,56]. However, given the numerous segments and qualifying variables involved, other multivariate analysis techniques become necessary. More recent research has turned to the Chi-square Automatic Interaction Detection (CHAID) algorithm as a method for market segmentation due to its increased sophistication [57] and its departure from the restrictive assumption of parametric testing for predictive variables. Although this method has found application in the health industry [58,59,60,61], its adoption in the tourism industry has been relatively less frequent.

Classification trees, such as those generated by the CHAID algorithm, encompass models in which the dependent variable is categorical, while regression trees involve continuous dependent variables [62]. The CHAID algorithm proves valuable in the realm of marketing segmentation as it can categorize variables of a categorical nature (e.g., country/place of origin, gender, accommodation, means of transportation used), which are prevalent in studies within this field. However, numerical continuous variables like age or income must be discretized into categorical variables before being employed in CHAID analysis.

The CHAID algorithm employs a criterion variable with multiple categories to facilitate segmentation based on this variable. It combines various independent variables, subject to the condition that Chi-square tests are statistically significant. Subsequently, it constructs nodes and defines segments, concluding when there is no significant relationship between the criterion variable and the explanatory variables. The most influential independent variable is typically presented in the initial node. Notably, CHAID proves more efficient in terms of variable number and data volume compared to other non-criteria methods, such as cluster analysis [63]. Consequently, this method allows policymakers in diverse tourism destinations to make more efficient and effective use of available information.

In this paper, an initial descriptive analysis was conducted to create a contingency table (cross-analysis) of key variables, such as accommodation type, transportation means used, visitors' place of residence, and more. Subsequently, the CHAID algorithm technique was employed to delineate the characteristics of different market segments. Adhering to the rule of thumb (or stopping rule) for tree growth [64], a minimum sample size for the concluding segments was considered in this study.

## 5 Results

### 5.1 Dependent variable: accommodation establishments

In accordance with Dolnicar's terminology [3], this study explores the interrelationships among "factors of travel" (Park, Frequent visitor, duration of stay, means of transport, accommodation, spend within) separately for the two destinations: Canary Islands and Florida. Since all the variables are categorical, we applied the Chi-square test after verifying that its basic assumptions were met. The findings are presented in Table 4.

**Table 4:** Relationship between Factors of Travel: Chi-square statistic and corresponding significance (p-value) in parentheses for Canary Islands (above the diagonal) and Florida (below the diagonal).

	PARK	FREQUENT_VISITOR	DURATION_ STAY	MEAN_ TRANSPORT	ACCOMMO-DATION	SPEND_ WITHIN
**PARK**		31.039 **(0.000∗)**	45.846 **(0.000∗)**	50.074 **(0.000∗)**	43.323 **(0.000∗)**	73.192 **(0.000∗)**
**FREQUENT_VISITOR**	10.294 **(0.006∗)**		23.128 **(0.000∗)**	182.667 **(0.000∗)**	244.064 **(0.000∗)**	22.867 **(0.000∗)**
**DURATION_STAY**	10.792 **(0.029∗)**	0.327 (0.849)		55.428 **(0.000∗)**	101.196 **(0.000∗)**	59.585 **(0.000∗)**
**MEAN_TRANSPORT**	24.554 **(0.002∗)**	9.287 **(0.026∗)**	4.602 (0.596)		448.361 **(0.000∗)**	37.006 **(0.000∗)**
**ACCOMMODATION**	9.845 (0.131)	13.462 **(0.004∗)**	40.171 **(0.000∗)**	28.841 **(0.001∗)**		46.693 **(0.000∗)**
**SPEND_WITHIN**	11.788 **(0.019∗)**	1.536 (0.464)	136.015 **(0.000∗)**	12.584 (0.050)	46.062 **(0.000∗)**	

∗ p-value <0.05 (Significative relationship).

From Tables 4 and it can be deduced that in the case of CNP, all the variables under consideration exhibit significant associations among them. However, for FSP, only 10 out of the 15 possible associations are statistically significant. Among these associations, the variables that display the strongest relationships with the others are "Park" and "Accommodation," both of which are linked to four of the remaining five variables. Notably, "Accommodation" stands out with the lowest p-values, indicative of higher Chi-squared values (supporting H1). Therefore, "Accommodation" has been selected as the dependent variable for both Florida and the Canary Islands in subsequent statistical analyses.

Additionally, when examining the level of influence on "Accommodation" from most to least significant (as determined by Chi-square and its corresponding significance), the top two variables for Canary Islands National Parks are "Mean_Transport_GR" and "Frequent_Visitor," while for Florida State Parks, it is "Spend_Within_GR" and "Duration_Stay_GR."

### 5.2 Results from the application of CHAID algorithm as a segmentation technique

From Tables 4 and it can be deduced that in the case of CNP, all the variables under consideration exhibit significant associations among them. However, for FSP, only 10 out of the 15 possible associations are statistically significant. Among these associations, the variables that display the strongest relationships with the others are "Park" and "Accommodation," both of which are linked to four of the remaining five variables. Notably, "Accommodation" stands out with the lowest p-values, indicative of higher Chi-squared values. Therefore, "Accommodation" has been selected as the dependent variable for both Florida and the Canary Islands in subsequent statistical analyses.

Additionally, when examining the level of influence on "Accommodation" from most to least significant (as determined by Chi-square and its corresponding significance), the top two variables for Canary Islands National Parks are "Mean_Transport_GR" and "Frequent_Visitor," while for Florida State Parks, it is "Spend_Within_GR" and "Duration_Stay_GR."

Only three out of the seven independent variables are shared between the two decision trees (refer to Table 5). It is noteworthy that the common independent variables in both decision trees, namely "Place-Residence," "Duration-Stay," and "Mean-Transport," appear in the final nodes of each classification. This finding further underscores the differences between the two resulting classifications (supporting H2) (see Table 6).

**Table 5:** Independent variables selected by the CHAID algorithm model to construct the trees for Canary Islands and for Florida.

Variables (Socio-demographic and factors of travel)	*CNP*	*FSP*
GENDER		
AGE		
**PLACE-RESIDENCE**	**X**	**X**
ANNUAL-INCOME		
STUDIES		
**OCCUPATION**		**X**
**NATIONAL/STATE PARK**	**X**	
**FREQUENT-VISITOR**	**X**	
**DURATION-STAY**	**X**	**X**
**MEAN-TRANSPORT**	**X**	**X**
**SPEND-WITHIN**		**X**

**Table 6:** Nodes (of the tree) associated with each category of ACCOMMODATION.

ACCOMMODATION^a^		(1)	(2)	(3)	(4)	Total
CNP	Ending nodes	12, 15, 16	5, 6, 10, 11	13, 18, 19, 20	17	
	n	385	289	167	22	863
	%	44.6 %	33.5 %	19.4 %	2.5 %	100 %
FSP	Ending nodes	4, 9, 10, 12, 13, 14, 16	11	15	–	
	n	830	18	93	–	941
	%	88.2 %	1.9 %	9.9 %	–	100 %

a Categories: (1) Hotel (2) Rented apartment, house or farmhouse (3) Own house or family/friend house (4) Camping, cruise and others.

**Fig. 1:**
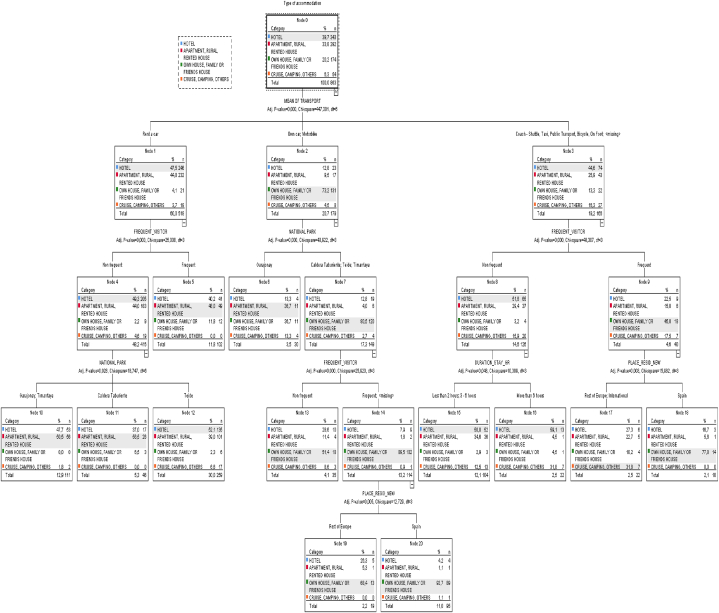
CANARY ISLANDS: CHAID tree for ACCOMMODATION_GR with 12 ending nodes. Stop criteria: Minimum cases in parent nodes (30).

**Fig. 2:**
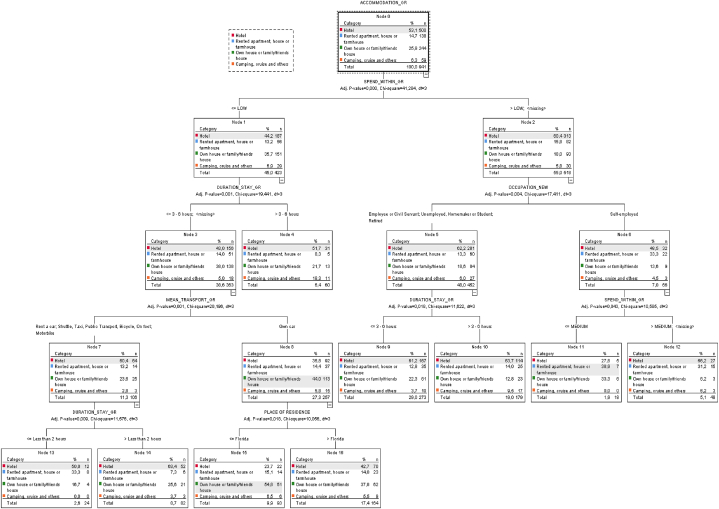
FLORIDA: CHAID tree for ACCOMMODATION_GR with 9 ending nodes. Stop criteria: Minimum cases in parent nodes (30).

When comparing the segmentations presented in Fig. 1, Fig. 2 based on the predominant "Accommodation" category in each terminal node of the respective tree, we observe that in CNP, the terminal nodes are more naturally aligned with the predominant categories. For instance, the group consisting of visitors who opt to stay in a Hotel (Category 1) is divided into three nodes or segments, accounting for 44.6 % of CNP visitors (in comparison to the observed 39.7 % in the sample). A similar pattern is observed for Categories 2 and 3 of "Accommodation," each divided into four nodes or segments that cover 33.5 % and 19.4 %, respectively (in comparison to the sample percentages of 33.8 % and 20.2 %).

In contrast, the segmentation in FSP is markedly different, with the predominant category in most of the obtained nodes or segments (7 out of 9) being Category 1, "Hotel," which accounts for 88.2%—significantly higher than the observed percentage in the sample (53.1 %). Consequently, Categories 2 and 3 with 1.9 % and 9.9 %, respectively, are represented only marginally in a single node or segment, and Category 4 is left unrepresented, in contrast to the CNP segmentation.

### 5.3 Online questionnaire vs face–to-face interviewing

After examining the divergent results obtained from segmenting the market of visitors to the parks in each region, it is essential to delve into the potential reasons behind this discrepancy. Relevant studies cited in the justification of the hypotheses shed light on this issue. The answer might lie in the motivations that drive visitors to participate in either an online or a face-to-face questionnaire. For instance, in the online survey conducted in Florida, a significant motivation could be the monetary incentive received for completing the questionnaire ($1). This economic motivation suggests that financial factors are likely to be significant, as evidenced by the presence of "spend within" and "occupation" in the initial nodes of the Florida State Parks (FSP) classification. In the Canary Islands, interviewers administered the face-to-face questionnaire to specific population groups without any economic selection criteria. However, various other motivations may be associated with the survey mode.

To compare the two modes of data collection, face-to-face and online, we will examine representativeness, data quality, and consistency following the methodology proposed by Dolnicar [3]. Initially, the representativeness of the two samples was compared using the socio-demographic profiles (refer to Table 1). These profiles reveal substantial differences between visitors to the natural parks of the Canary Islands and Florida. The significance (p-values) obtained for the Chi-square statistics in Table 1 confirms significant disparities between the two destinations for all socio-demographic variables (p < 0.05), except for Gender and Place_Residence. Particularly noteworthy differences in the profiles of visitors between the two destinations, concerning socio-demographic variables, are observed in the following areas:

AGE_GR: A striking difference is evident in the age distribution, with 4.2 % of visitors to FSP being over 55 years old, compared to 33.9 % of visitors to the Canary Islands National Park (CNP) within the same age range.

STUDIES: Educational attainment varies significantly, with 85.5 % of FSP visitors having a university education compared to 54.7 % of CNP visitors.

ANNUAL_INCOME: The income distribution shows considerable disparities, as the low-income bracket accounts for 54.4 % of the FSP sample and only 26.6 % for the CNP sample.

These observed differences in income and age profiles between visitors from the two populations, particularly the much lower income and older age of the Florida online sample, likely explain the higher significance of economic variables ("Spend-Within" and "Occupation") in the Florida tree compared to the Canary Islands classification. In fact, these economic variables are not significant for the CNP.

Secondly, data quality is assessed using two criteria: the non-response percentage and contamination. The non-response percentage, which indicates low data quality, is the percentage of unanswered questions. In our case, the non-response rate is 14.3 % for FSP (primarily among visitors aged 55 and over) and only 0.2 % for CNP, signifying a higher response rate for CNP. Contamination, resulting from different response styles, is another indicator of low data quality. To evaluate this, we consider 17 survey variables that assess various aspects of park services, such as restaurants, cafes, visitor centers, accommodation, shops, security, maintenance, vehicles, guides, shelters, information, other services, parking, signposting, paths, accessibility, and other facilities. These variables employ a five-point Likert scale: (1) Very bad, (2) Bad, (3) Fair, (4) Good, (5) Very good (see Table 3).

Upon initial examination and comparison of the surveys conducted in CNP and FSP, as detailed in Table 7, it appears that there are no significant differences in the response style indicators, except for ERS+, using the non-parametric Mann-Whitney test, as the basic assumptions of the corresponding parametric test were not met. This suggests that there are no major disparities in terms of data contamination between the two surveys. It is noteworthy that both ERS+ and ARS indicators have high average values. For ERS+, it indicates that 35.9 % of CNP visitors and 40.2 % of FSP visitors frequently choose the response (5), with Florida showing significantly higher values. Additionally, both ERS+ and ARS exhibit the highest dispersion among all the calculated indicators, with standard deviations of 0.322 for CNP and 0.303 for FSP. However, the percentages of choosing responses (4) or (5), which represent ARS, are quite similar, at 74.3 % for CNP and 76.7 % for FSP. These findings underscore the significant value that visitors in both destinations place on the protected natural areas under study.

**Table 7:** Response style difference between CNP and FSP for 17 rating variables (mean and standard deviation in parenthesis).

	ERS -	DRS	MRS	ARS	ERS +
CNP	**0.017 (0.074)**	**0.066 (0.144)**	**0.191 (0.249)**	**0.743 (0.277)**	**0.359 (0.322)**
FSP	**0.018 (0.075)**	**0.066 (0.146)**	**0.167 (0.179)**	**0.767 (0.250)**	**0.402 (0.303)**
Means comparison p-value	0.715	0.988	0.992	0.993	**0.003∗**

∗ p-value <0.05 (Significant differences).

Specifically, ERS+ was calculated for various levels of these socio-demographic variables, such as place of residence, age, and education, resulting in the outcomes presented in Table 8. When comparing ERS + values in the Canary Islands and Florida across categories of these three variables, significant differences emerge. In terms of age groups, visitors aged 36–55 and over 55 tend to choose the (5) option more frequently in FSP than in CNP. For place of residence categories, significant differences only exist among visitors from the respective countries, indicating that those residing in Florida are more inclined to select the (5) option compared to visitors residing in Spain. Regarding education, significant differences are observed solely in the third category (Degree or postgraduate degree), suggesting that visitors with the highest level of education tend to choose the highest option more frequently in FSP than in CNP.

**Table 8:** ERS + for age, place of residence (separating the categories of Rest of Europe/USA and International) and studies, categorized as (1), (2), and (3).

Categories →	(1)			(2)			(3)		
Means	CNP	FSP	p-value	CNP	FSP	p-value	CNP	FSP	p-value
**AGE**	0.377	0.390	0.549	0.351	0.435	**0.001**^a^	0.353	0.446	**0.044**^a^
**PLACE_RES.**	0.331	0.412	**0.001**^a^	0.377	0.397	0.266	0.353	0.398	0.642
**STUDIES**	0.432	0.368	0.471	0.362	0.359	0.904	0.349	0.409	**0.001**^a^

a Significant differences.

In the final phase of this study, we turned our attention to comparing the consistency of data by analyzing the relationships among the travel factors outlined in Table 2. The corresponding significance (p-value) for the chi-square statistic for CNP and FSP is presented in this table. The analysis revealed significant differences in visitor behavior between CNP and FSP across travel factors, except for Duration_Stay and Spend_Within.

For Duration_Stay, it was observed that in both cases, more than 60 % of visitors spent between 3 and 6 h during their visit. As for Spend_Within, approximately 50 % of visitors in both destinations had a low expenditure level within the park.

Furthermore, we compared the visitor profiles in both destinations, highlighting key distinctions in Mean_Transport_GR and Accommodation_GR. For Mean_Transport_GR, in CNP, the dominant choice was Rent a car (60.6 %), whereas in FSP, Own car prevailed (65.3 %). Regarding Accommodation_GR, differences were also evident. In CNP, Hotel was the preferred option for visitors (39.7 %), followed closely by Rented apartment, house, or farmhouse (33.8 %). In contrast, FSP visitors overwhelmingly favored Hotel (53.1 %) compared to Rented apartments (14.7 %). The percentages for Own house or family/friend house were similar (20.2 % for CNP and 25.9 % in FSP).

Based on these comparisons, it can be concluded that significant differences exist in the socio-demographic profiles of visitors in the two samples, with CNP, the face-to-face survey, being more representative than FSP, the online survey (supporting H3). Additionally, differences were observed in visitor behavior with regard to travel factors (H6).

Moreover, we delved into the issue of data quality, analyzing the number of omissions (non-responses to questions) and contamination by response styles. A higher percentage of non-responses was observed in the online survey (H4). However, this discrepancy did not lead to significant differences in data contamination between the two samples, except for ERS+, which yielded interesting results when examined in detail, showing that certain categories of demographic variables (place of residence, age, or studies) have a significant influence on ERS+ (H5).

## 6 Discussion

In this study, it is important to note that relatively few papers explore the segmentation of visitors to national and state parks [65,66]. Even fewer studies compare the market segmentation of park visitors across different territories. Notably, no previous study has employed the CHAID algorithm method for this analysis. Additionally, as Liu et al. point out, while there is ample literature on information collection methods and response styles, it is rare for research to examine these two aspects concurrently [28]. Therefore, the findings of Liu et al. are comparable to those of this study, though there are notable differences concerning ERS + that merit attention for their contribution to the literature [28].

The consistency of results between this study and those of Liu et al. after a decade suggests that Likert scales can still be used in online surveys without introducing significant bias compared to face-to-face methods [28]. The notable exception, according to our findings, is ERS+. This study explores ERS + more thoroughly than previously, marking the first instance in the literature where a study of this nature has identified significant differences between the two survey modes in terms of socioeconomic variables such as age, place of residence, and educational level.

However, it is important to consider that our analysis was conducted in two culturally distinct countries and involved compensating online survey respondents with one US dollar. Further research is needed to explore how these factors influence the age demographics of respondents in both populations, as well as their economic and educational levels. For instance, a one-dollar compensation might be more appealing to younger, university-going individuals who typically have fewer economic resources, compared to middle-aged individuals. In the Canary Islands, face-to-face surveys were administered to specific population groups without any economic selection criteria.

The analysis includes a thorough examination of representativeness, data quality (encompassing both encompassing and contamination), and consistency. The findings correlate with prior literature, such as Dillman et al., which noted a tendency for respondents to select extreme values in online questionnaires [2]. Although the two populations surveyed in our study differ, the levels of contamination appear similar, suggesting that contamination persists across different samples, akin to Dolnicar's observations in studies using a multi-mode system with the same population [3]. In our study, this tendency towards extreme positive valuation (higher ratings) was particularly notable. Furthermore, a detailed analysis of ERS + considering demographic variables (age, place of residence, educational level) shows significant differences between the two tourist destinations, the Canary Islands and Florida. Additionally, despite Dolnicar suggesting that offering different response modes might mitigate these issues [3], our study demonstrates that the "mode effect" persists even when respondents are provided with different modes of response.

This study, which focuses on selecting the optimal survey mode, carries significant implications for survey research, particularly in the realm of nature tourism. Although multi-mode surveys are often recommended in the literature, especially for identical populations, our research examined a different scenario involving distinct populations assessing the same tourism product. Our study included two separate statistical populations in different countries, surveyed at different times to minimize the mode effect. We used the same questionnaire for both groups, with one surveyed online and the other face-to-face. Our findings indicate that the mode effect persists even when considering different populations located in diverse geographical and socio-cultural environments and surveyed at different times. The analysis provides a comprehensive assessment of data quality, including non-responses, representativeness, factors affecting responses, and consistency.

It is noteworthy that despite a smaller sample error of 0.50, the online sample exhibited lower representativeness than the face-to-face survey across sociodemographic variables and travel factors. Additionally, the online survey recorded a higher percentage of non-responses. However, there was no significant difference in contamination levels between the two samples, except for ERS+ (Extreme Response Style). A detailed examination of ERS + revealed that certain demographic variables (place of residence, age, or education) significantly influence this response style in both models. Moreover, when comparing the ERS + values between Spain and Florida and considering different categories of these variables, significant differences emerged.

Liu et al. conducted a study in 2012 similar to ours, administering the same questionnaire through both face-to-face interviews and web surveys to two independent national probability samples within the same country [28]. In contrast, our study spans two different countries. Similarly, our results also relate in some ways to Zhang et al. [1], which analyzes cultural differences between countries. However, Zhang et al. employed the same data collection method in both countries, making it less effective for assessing the mode effect [1].

In summary, this empirical study compares the results of administering the same questionnaire in different countries using different data collection methods, with representative samples of the population, to assess the mode effect. The findings demonstrate that the mode effect continues to persist.

## 7 Conclusion

### 7.1 Summary of the research, key findings, and theoretical contributions

This study makes a significant contribution to the existing literature by addressing the recommendation of employing multi-mode surveys to mitigate the "mode effect" [2,3,34,1]. Our statistical analysis demonstrates that despite our efforts to neutralize this "mode effect" by utilizing two distinct populations for the same tourism product to prevent any biases related to visitor preferences for one survey method over the other, the face-to-face procedure exhibits markedly higher levels of representativeness, data quality, and consistency compared to the online method. This is evidenced by the confirmation of hypotheses H3, H4, and H5. Furthermore, hypotheses H2 and H6 are validated, as significant differences in visitor behavior have been identified across the variables considered. These differences are crucial both for describing visitor profiles and for their use as predictors in segmentation analysis using CHAID, where accommodation serves as the dependent variable due to its strong relationship with other variables, as suggested by H1.

### 7.2 Practical contributions

Considering the recent COVID-19 crisis, which made face-to-face surveys challenging, we recommend the following strategies: 1) Implement programmed filters in online surveys to ensure a quota-based system for each sociological variable, enhancing representativeness; 2) Explore ways to mitigate the monetary effect of incentives (e.g., increasing the incentive amount) to encourage responses from a broader visitor demographic; 3) Consider combining online surveys with phone surveys, especially for groups less familiar with the internet, such as those over 55 or individuals with lower educational levels; therefore phone surveys can complement online surveys to bridge potential gaps in the sample.

### 7.3 Study limitations and future research

The sample obtained through MTurk is often convenient but may not be representative of the broader population. With MTurk, researchers typically post tasks or surveys, and respondents choose whether or not to participate. This self-selection process inherently introduces bias and makes it difficult to ensure a truly random sample. Without robust verification mechanisms, there's a risk of including participants who falsely claim to have visited the parks, leading to inaccurate data.

Given these considerations, while MTurk can be a useful tool for certain research purposes, it may not be the most suitable option for obtaining a random sample of park visitors. Alternative methods, such as onsite surveys or systematic sampling techniques, may be more effective for this particular research goal.

In our case, there is a lack of greater participation of individuals aged 55 and over in the sample obtained through Mturk, in which younger volunteers usually participate. However, it should be noted that, out of the 14.3 % of non-responses or incomplete responses to the questionnaire questions, a large portion belongs to this age group.

## CRediT authorship contribution statement

**Flora M. Díaz-Pérez:** Writing – original draft. **Alan Fyall:** Writing – review & editing. **Carlos Gustavo García-González:** Writing – original draft. **Xiaoxiao Fu:** Writing – review & editing. **Gary Deel:** Writing – original draft.

## Data and code availability statement

Data will be made available on request.

## Declaration of competing interest

The authors declare that they have no known competing financial interests or personal relationships that could have appeared to influence the work reported in this paper.
