# Supplementary material for: Navigating the "mode effect": A comparison of online questionnaires and face-to-face interviews
Source: Heliyon. 2025 Jan 6;11(1):e41742. doi: 10.1016/j.heliyon.2025.e41742 (PMC11762187; doi:10.1016/j.heliyon.2025.e41742)
Supplement: Multimedia component 1 [file mmc1.docx]

QUESTIONNAIRE

Questionnaire number

|  |  |  |
| --- | --- | --- |

**1. -Have you visited at least one of the following State Park(s) in the past three years? Please indicate yes or no.**

1. Florida Keys Overseas Heritage Trail

2. Honeymoon Island State Park

3. Marjorie Harris Carr Cross Florida Greenway

**2. – Which one(s) have you visited in the past three years? Check all that apply.**

1. Florida Keys Overseas Heritage Trail

2. Honeymoon Island State Park

3. Marjorie Harris Carr Cross Florida Greenway

**3. - Could you tell us your usual place of residence?**

| 1 | Florida |
| --- | --- |
| 2 | U.S. other than Florida |
| 3 | International, please specify |

**4.- Was your most recent visit to one of the three State Parks in Florida your first ever visit to that park?**

| 1 | It is the first time |
| --- | --- |
| 2 | It is the second time |
| 3 | It is the third time |
| 4 | More than three times |
| 5 | Visit Frequently |

**5. - How many State Parks in Florida have you visited in total? Please fill in the blank.**

**6.- Do you normally visit the State Parks in Florida alone or with company?**

| 1 | Alone |
| --- | --- |
| 2 | With your partner |
| 3 | With your family |
| 4 | With friends |
| 5 | Part of an organized trip in a group larger than 8 people but less than 15 |
| 6 | Part of an organized trip in a group larger than 15 people but less than 25 |
| 7 | Part of an organized trip in a group larger than 25 people |

**7. -Could you mark the services you have experienced in the following State Park(s)?**

|  | **Services** | **Florida Keys Overseas Heritage Trail** | **Honeymoon Island State Park** | **Marjorie Harris Carr Cross Florida Greenway** |
| --- | --- | --- | --- | --- |
| 1 | Restaurants |  |  |  |
| 2 | Cafés and bars |  |  |  |
| 3 | Visitors’ Centre |  |  |  |
| 4 | Accommodation |  |  |  |
| 5 | Shops and other commercial establishments |  |  |  |
| 6 | Security and supervision services |  |  |  |
| 7 | State Park maintenance services |  |  |  |
| 8 | Park vehicles |  |  |  |
| 9 | State Park Guides |  |  |  |
| 10 | Shelter |  |  |  |
| 11 | Park Information Services |  |  |  |
| 12 | Others |  |  |  |

**8. -How would you rate the services in the following State Park(s), on a scale of 1 to 5, with 1 being “Very Bad” and 5 “Very Good”?**

|  | **Services** | **Florida Keys Overseas Heritage Trail** | | | | | **Honeymoon Island State Park** | | | | | **Marjorie Harris Carr Cross Florida Greenway** | | | | |
| --- | --- | --- | --- | --- | --- | --- | --- | --- | --- | --- | --- | --- | --- | --- | --- | --- |
|  |  | **1** | **2** | **3** | **4** | **5** | **1** | **2** | **3** | **4** | **5** | **1** | **2** | **3** | **4** | **5** |
| 1 | Restaurants |  |  |  |  |  |  |  |  |  |  |  |  |  |  |  |
| 2 | Cafés and bars |  |  |  |  |  |  |  |  |  |  |  |  |  |  |  |
| 3 | Visitors’ Centre |  |  |  |  |  |  |  |  |  |  |  |  |  |  |  |
| 4 | Accommodation |  |  |  |  |  |  |  |  |  |  |  |  |  |  |  |
| 5 | Shops and other commercial establishments |  |  |  |  |  |  |  |  |  |  |  |  |  |  |  |
| 6 | Security and supervision services |  |  |  |  |  |  |  |  |  |  |  |  |  |  |  |
| 7 | State Park maintenance services |  |  |  |  |  |  |  |  |  |  |  |  |  |  |  |
| 8 | Park vehicle |  |  |  |  |  |  |  |  |  |  |  |  |  |  |  |
| 9 | State Park Guides |  |  |  |  |  |  |  |  |  |  |  |  |  |  |  |
| 10 | Shelter |  |  |  |  |  |  |  |  |  |  |  |  |  |  |  |
| 11 | Park Information Services |  |  |  |  |  |  |  |  |  |  |  |  |  |  |  |
| 12 | Others |  |  |  |  |  |  |  |  |  |  |  |  |  |  |  |

Facilities

**9. -Have you experienced the facilities in the following State Park(s)?**

|  | **Facilities** | **Florida Keys Overseas Heritage Trail** | **Honeymoon Island State Park** | **Marjorie Harris Carr Cross Florida Greenway** |
| --- | --- | --- | --- | --- |
| 1 | Parking |  |  |  |
| 2 | Signposting (information boards, road markings, places, etc.) |  |  |  |
| 3 | Signposts for paths |  |  |  |
| 4 | Accessibility (Visitors with limited mobility) |  |  |  |
| 5 | Others |  |  |  |

**10. -How would you rate the following facilities in the following State Park(s), on a scale of 1 to 5, with 1 being “Very Bad” and 5 “Very Good”?**

|  | **Facilities** | **Florida Keys Overseas Heritage Trail** | | | | | **Honeymoon Island State Park** | | | | | **Marjorie Harris Carr Cross Florida Greenway** | | | | |
| --- | --- | --- | --- | --- | --- | --- | --- | --- | --- | --- | --- | --- | --- | --- | --- | --- |
|  |  | **1** | **2** | **3** | **4** | **5** | **1** | **2** | **3** | **4** | **5** | **1** | **2** | **3** | **4** | **5** |
| 1 | Parking |  |  |  |  |  |  |  |  |  |  |  |  |  |  |  |
| 2 | Signposting (information boards, road markings, places etc). |  |  |  |  |  |  |  |  |  |  |  |  |  |  |  |
| 3 | Signposts for paths |  |  |  |  |  |  |  |  |  |  |  |  |  |  |  |
| 4 | Accessibility (Visitors with limited mobility) |  |  |  |  |  |  |  |  |  |  |  |  |  |  |  |
| 5 | Others |  |  |  |  |  |  |  |  |  |  |  |  |  |  |  |

**11. -Considering the services that are already offered in the following State Park(s), what other services do you think the park(s) could add to its offer?**

| 1 | Services | **Florida Keys Overseas Heritage Trail** | **Honeymoon Island State Park** | **Marjorie Harris Carr Cross Florida Greenway** |
| --- | --- | --- | --- | --- |
|  |  |  |  |  |

**12. -Could you indicate (on a scale from 1 to 10) to what extent you consider the natural resources of the State Park(s) have been preserved? With 1 being “Very Bad” and 10 “Very Good”**

|  | 1 | 2 | 3 | 4 | 5 | 6 | 7 | 8 | 9 | 10 |
| --- | --- | --- | --- | --- | --- | --- | --- | --- | --- | --- |
| **Florida Keys Overseas Heritage Trail** |  |  |  |  |  |  |  |  |  |  |
| **Honeymoon Island State Park** |  |  |  |  |  |  |  |  |  |  |
| **Marjorie Harris Carr Cross Florida Greenway** |  |  |  |  |  |  |  |  |  |  |

**13.- Answer only if you also visited the State Park(s) before the outbreak of Covid-19. Rate on a scale of 1 to 5 the state of conservation of natural resources in the State Park(s)**:

|  | 1 Badly preserved | 2 Preserved but not to previous standard(s) | 3 Equally preserved | 4 Better preserved | 5 Much better preserved than before the outbreak of Covid-19 |
| --- | --- | --- | --- | --- | --- |
| **Florida Keys Overseas Heritage Trail** |  |  |  |  |  |
| **Honeymoon Island State Park** |  |  |  |  |  |
| **Marjorie Harris Carr Cross Florida Greenway** |  |  |  |  |  |

**14.- How has Covid-19 impacted your enjoyment of the natural resources in the State Park(s)?**

1 Not impacted my enjoyment of the natural resources at all

2 Impacted my enjoyment of the natural resources only a little

3 My enjoyment of natural resources has been the same as before

4 Very much impacted my enjoyment of the natural resources

5 It has impacted my enjoyment of natural resources to a great extent

**15.- Since the outbreak of Covid-19, are you more likely to frequent State Parks than before?**

1 Yes

2 No

**16.- (ONLY ANSWER THIS QUESTION IF YOUR ANSWER TO QUESTION 15 WAS YES) To what extent was your staying in the park more impactful than before Covid19?**

1 Not impacting my stay at all

2 Impacting only a little

3 It impacted the same

4 Very much impacting

5 It has impacted my stay to a great extent

**17. -What transportation do you normally use to reach the State Park(s)?**

|  | Transportation | **Florida Keys Overseas Heritage Trail** | **Honeymoon Island State Park** | **Marjorie Harris Carr Cross Florida Greenway** |
| --- | --- | --- | --- | --- |
| 1 | Own car |  |  |  |
| 2 | Rent-a-car |  |  |  |
| 3 | Taxi |  |  |  |
| 4 | Shuttle |  |  |  |
| 5 | Bicycle |  |  |  |
| 6 | Motorbike |  |  |  |
| 8 | On foot |  |  |  |
| 9 | Others |  |  |  |

**18. -On your journey to and from the park(s), how much did you spend on each of the following services (activities), products (in shops), etc.? Please indicate the approximate spending:**

|  | Services (in dollars) | **Florida Keys Overseas Heritage Trail** | **Honeymoon Island State Park** | **Marjorie Harris Carr Cross Florida Greenway** |
| --- | --- | --- | --- | --- |
| 1 | Restaurants |  |  |  |
| 2 | Cafés and bars |  |  |  |
| 3 | Public Transport |  |  |  |
| 4 | Coach |  |  |  |
| 5 | Shuttle |  |  |  |
| 6 | Taxi |  |  |  |
| 7 | Rent-a-car, per day |  |  |  |
| 8 | Fuel (petrol, diesel, etc.) |  |  |  |
| 9 | Guide |  |  |  |
| 10 | Tour (cost per day) |  |  |  |
| 11 | Others |  |  |  |

**19. -What was your approximate spending inside the State Park(s)?**

|  |  | **Florida Keys Overseas Heritage Trail** | **Honeymoon Island State Park** | **Marjorie Harris Carr Cross Florida Greenway** |
| --- | --- | --- | --- | --- |
| 1 | Restaurants |  |  |  |
| 2 | Cafés and bars |  |  |  |
| 3 | Shopping |  |  |  |
| 4 | Guides |  |  |  |
| 5 | Accommodation |  |  |  |
| 6 | Park vehicle |  |  |  |
| 7 | Others |  |  |  |

**20. -How long did your visit take inside the State Park?**

|  | Less than 1 hour | between 1 and 2 hours | between 2 and 4 hours | between 4 and 6 hours | More than 6 hours | More than a day |
| --- | --- | --- | --- | --- | --- | --- |
| **Florida Keys Overseas Heritage Trail** |  |  |  |  |  |  |
| **Honeymoon Island State Park** |  |  |  |  |  |  |
| **Marjorie Harris Carr Cross Florida Greenway** |  |  |  |  |  |  |

**Personal Data**

**21. –Gender**

| 1 | Male |
| --- | --- |
| 2 | Female |

**22. -Age**

| 1 | 18-25 |
| --- | --- |
| 2 | 26 – 35 |
| 3 | 36 – 45 |
| 4 | 46 – 55 |
| 5 | 55 – 65 |
| 6 | + 65 |

**23. -Marital**

| 1 | Single |
| --- | --- |
| 2 | Married |
| 3 | Divorced |
| 4 | Widow/er |

**24. -Current employment**

| 1 | Homemaker |
| --- | --- |
| 2 | Unemployed |
| 3 | Retired |
| 4 | Student |
| 5 | Civil Servant |
| 6 | Self-employed |
| 7 | Employee |

**25. -Studies completed**

| 1 | Less than high school |
| --- | --- |
| 2 | High school incomplete |
| 3 | High school diploma |
| 4 | Associate degree |
| 5 | Bachelor’s degree |
| 6 | Postgraduate degree |

**26. -Annual income of household**

| 0 | Don’t know/No comment |
| --- | --- |
| 1 | Less than $9,000 |
| 2 | Between $9001 and $15.000 |
| 3 | Between $15,001 and $24,000 |
| 4 | Between $24,001 and $35,000 |
| 5 | Between $35,001 and $50,000 |
| 6 | Between $50,001 and $75,000 |
| 7 | Between $75,001 and $150,000 |
| 8 | More than $150,000 |

**27. -If staying outside of your home, where did you stay when visiting the State Park(s)?**

|  | **Accommodation** | **Florida Keys Overseas Heritage Trail** | **Honeymoon Island State Park** | **Marjorie Harris Carr Cross Florida Greenway** |
| --- | --- | --- | --- | --- |
| 1 | Hotel with 3 stars or fewer |  |  |  |
| 2 | Hotel with 4 stars |  |  |  |
| 3 | Hotel with 5 or more stars |  |  |  |
| 4 | Apartment |  |  |  |
| 5 | Rural cottage/farmhouse |  |  |  |
| 6 | Rented house |  |  |  |
| 7 | Camping, tent |  |  |  |
| 8 | Family or friends house |  |  |  |
| 9 | Cruise |  |  |  |
| 10 | Others (Specify) |  |  |  |
| 11 | Own house(residence) |  |  |  |

**28.- What is your zip code?**

**======================End of survey. Thank you for your participation================**
